# Analysis of a large cohort of cystic fibrosis patients with severe liver disease indicates lung function decline does not significantly differ from that of the general cystic fibrosis population

**DOI:** 10.1371/journal.pone.0205257

**Published:** 2018-10-11

**Authors:** Deepika Polineni, Annalisa V. Piccorelli, William B. Hannah, Sarah N. Dalrymple, Rhonda G. Pace, Peter R. Durie, Simon C. Ling, Michael R. Knowles, Jaclyn R. Stonebraker

**Affiliations:** 1 School of Medicine, Marsico Lung Institute, UNC Cystic Fibrosis Research Center, The University of North Carolina at Chapel Hill, Chapel Hill, North Carolina, United States of America; 2 Department of Pulmonology, Division of Pulmonary and Critical Care Medicine, School of Medicine, The University of Kansas, Kansas City, Kansas, United States of America; 3 Department of Epidemiology and Biostatistics, School of Medicine, Case Western Reserve University, Cleveland, Ohio, United States of America; 4 Department of Mathematics and Statistics, University of Wyoming, Laramie, Wyoming, United States of America; 5 Physiology and Experimental Medicine, Research Institute, The Hospital for Sick Children, Toronto, Ontario, Canada; 6 Division of Gastroenterology, Hepatology and Nutrition, The Hospital for Sick Children, University of Toronto, Toronto, Ontario, Canada; 7 Department of Paediatrics, University of Toronto, Toronto, Ontario, Canada; University of Alabama-Birmingham, UNITED STATES

## Abstract

Previous reports of lung function in cystic fibrosis (CF) patients with liver disease have shown worse, similar, or even better forced expiratory volume in 1 second (FEV_1_), compared to CF patients without liver disease. Varying definitions of CF liver disease likely contribute to these inconsistent relationships reported between CF lung function and liver disease. We retrospectively evaluated spirometric data in 179 subjects (62% male; 58% Phe508del homozygous) with severe CF liver disease (CFLD; defined by presence of portal hypertension due to cirrhosis). FEV_1_ values were referenced to both a normal population (FEV_1_% predicted) and CF population (CF-specific FEV_1_ percentile). We utilized a linear mixed model with repeated measures to assess changes in lung function (before and after diagnosis of CFLD), relative to both the normal and CF populations. At diagnosis of CFLD, the mean FEV_1_ was 81% predicted, or at the 53^rd^ percentile referenced to CF patients without CFLD. There was a significant difference in post-CFLD slope compared to pre-CFLD slope (post–pre) using FEV_1_% predicted (-1.94, p-value < 0.0001). However, there was insignificant evidence of this difference using the CF-specific FEV_1_ percentile measure (-0.99, p-value = 0.1268). Although FEV_1_% predicted values declined in patients following CFLD diagnosis, there was not significant evidence of lung function decline in CF-specific FEV_1_ percentiles. Thus, the observed study cohort indicates diagnosis of severe CFLD was not associated with worsened CF lung disease when compared to a large CF reference population.

## Introduction

Cystic fibrosis (CF) is the most common lethal genetic disorder in Caucasians, affecting an estimated 30,000 persons in the United States [1]. Mutations in the cystic fibrosis transmembrane conductance regulator (*CFTR*) gene cause abnormal ion transport at the apical membrane of airway epithelial cells, resulting in hyperviscous airway mucus [2, 3], and similar pathophysiology may occur in additional organs. Severe CF liver disease (CFLD) with portal hypertension due to cirrhosis is the most common non-pulmonary cause of mortality in CF (after pulmonary disease and transplantation complications) [4–6]. While many CF patients have some degree of liver abnormality, only 5% develop severe CF liver disease with portal hypertension (CFLD) due to cirrhosis [7]. Severity of CF lung disease, and decline in lung function, reflect both heritable and environmental factors [8, 9], and the presence of CFLD has been hypothesized to influence lung function.

The relationship between lung function and liver disease in CF has been unclear, however, as previous studies have reported variable results. For example, studies have reported that CF patients with liver disease have milder lung disease than those without liver disease [10, 11], and there is a slower deterioration of FEV_1_ in subjects with liver disease compared to matched CF controls [12]. However, other case-control studies report findings of worse FEV_1_ in children with liver disease compared to those without [13, 14]. In contrast, a prospective study of the impact of hepatic disease on the clinical course of CF subjects followed over a median 14 years showed no significant difference between subjects with or without CF liver disease in the frequency of FEV_1_% predicted <60% [15], which is congruent with additional studies showing no correlation between liver disease and lung disease [16, 17]. Taken together, the divergent findings on the relationship between hepatic disease and lung function may be due to variable definitions of CF liver disease.

To explore the relationship between lung function and liver disease in CF, we elected to analyze patients with an extreme phenotype of CF liver disease (severe CFLD; defined by portal hypertension and cirrhosis), to validate the presence of CFLD in the study population. We have previously identified and characterized a cohort of rigorously phenotyped severe CFLD patients, providing a detailed description of severe liver disease in the CF population [18]. In a subset of these severe CFLD patients from the United States and Canada with spirometric data, we analyzed FEV_1_ measurements 1) before, 2) at (or immediately prior to), and 3) after diagnosis of CFLD to determine the relationship between severe CFLD and lung function. Because it is well known that there is substantial lung function decline in adolescents and young adults in CF (but not in normal populations), in addition to FEV_1_% predicted, we used a CF-population specific FEV_1_ measure to define lung function.

## Methods

### Study design and participants

Severe CFLD subjects described in this analysis were enrolled in the Genetic Modifier Study of Severe CF Liver Disease from 34 North American CF Centers from the United States (n = 145 CFLD patients) and Canada (n = 34 CFLD patients), with a coordinating center at The University of North Carolina at Chapel Hill as previously described [7]. The respective Institutional Review Boards of all participating institutions approved this study, and are listed in S1 Table. All subjects provided written informed consent (subjects ≥ 18 years old) or assent (subjects < 18 years old); and, informed consent was obtained from subjects’ parents or guardian for subjects < 18 years old.

### Inclusion/Exclusion criteria

All subjects had a diagnosis of CF confirmed by sweat chloride test and/or *CFTR* genotyping. Diagnosis of severe CFLD was confirmed among subjects two years or older by 1) imaging evidence of hepatic parenchymal abnormalities (ultrasound, computed tomography, or magnetic resonance imaging) consistent with cirrhosis (including heterogeneous liver parenchyma), and 2) evidence of portal hypertension (esophageal varices at endoscopy, portosystemic collateral vascularization on imaging, or splenomegaly determined by physical exam or imaging), with no alternative diagnosis for liver disease or portal hypertension present. Pulmonary function tests (PFTs) post-liver transplant in CFLD patients were not included in the analysis. No subjects were excluded due to their self-defined race.

### Validation of CFLD study criteria

Case report form data (obtained from subjects’ medical records by the CF coordinator or principal investigator at each center) and source data (i.e. subjects’ radiology, endoscopy, and clinical records), were independently reviewed by two physicians with expertise in severe CFLD (Drs. Peter Durie and Simon Ling), to ensure inclusion and exclusion criteria were strictly met.

### Lung function

We retrospectively examined spirometry data (FEV_1_% predicted and CF-specific FEV_1_ percentile) [19, 20] in a cohort of 179 subjects with severe CFLD as defined above. FEV_1_ measurements were obtained from spirometry measures performed in subjects ≥ six years old. All available FEV_1_ measures recorded prior to and/or after CFLD diagnosis were captured for analysis. In cases of spirometric testing pre- and post- administration of a short acting bronchodilator, only the pre-bronchodilator values were retained for analysis. FEV_1_ values, measured in liters (L), were referenced to a normal (non-CF, healthy) population [19] when calculating the FEV_1_% predicted. To analyze lung function, and lung function decline relative to patients with CF, we utilized a disease specific equation [20] to reference FEV_1_ measures to a CF population matched by age, gender, and height, and calculated CF-specific FEV_1_ percentiles.

### Statistical analysis

Data were analyzed in subjects with FEV_1_ measures pre- and post- diagnosis of severe CFLD (Pre-/Post- cohort). We further performed analysis including those patients who only had FEV_1_ measures post-severe CFLD diagnosis (Post cohort) (S2 Table). For the Pre-/Post- cohort, lung function was analyzed to model the slope of lung function decline, and to compare the slope of decline pre- versus post-severe CFLD diagnosis. The Pre-/Post- cohort data were analyzed using a piecewise linear mixed model, which assumed that the lung function measurement (either FEV_1_% predicted, or CF-specific FEV_1_ percentile) followed a linear regression on time (years) before or after diagnosis of CFLD, where the slope could be different before versus after diagnosis of severe CFLD. The Post cohort was also analyzed to model the rate of lung function decline after severe CFLD diagnosis, including an additional 61 subjects with post-severe CFLD diagnosis lung function measures. The Post cohort data were analyzed with a linear mixed model, which assumed that the lung function measurement (either FEV_1_% predicted, or CF-specific FEV_1_ percentile) followed a linear regression on time (years) after severe CFLD diagnosis. The models of both the Pre-/Post- cohort and the Post cohort included age of severe CFLD diagnosis as a covariate, as well as the interaction between age of severe CFLD diagnosis and time of PFTs relative to severe CFLD diagnosis (i.e., time before CFLD for the Pre-/Post- cohort and time after CFLD for the Post cohort). Subject-specific random effects for intercept and pre- and post-severe CFLD diagnosis slopes for the Pre-/Post- cohort, or post-severe CFLD diagnosis slope for the Post cohort, were included in the model. The model assumed an unstructured covariance for the 2 x 2 covariance matrix of the random intercept and slope. In the linear mixed model analyses we tested whether pre- and post-CFLD slopes differed from zero in both cohorts, and whether pre- and post-CFLD slopes differed from each other in the Pre-/Post- cohort. Residual plots of the linear mixed models were assessed to determine influential outliers according a Cook’s Distance boundary >4/n; based on this criterion, no influential observations were found in either cohort.

### Role of the funding source

The sponsors of the study had no role in the study design, data collection, analysis and interpretation, writing of the manuscript, or decision to submit the manuscript for publication. The corresponding author had full access to all study data and bears final responsibility for the decision to submit for publication.

## Results

We studied a cohort of 179 subjects with severe CFLD (62% male; 58% Phe508del homozygous), with 8,249 measures of spirometry (1,840 pre-diagnosis, 6,409 post-diagnosis measures) (Table 1). In a subset of this cohort, 118 subjects had spirometry data captured both pre- and post-severe CFLD diagnosis (Pre-/Post- cohort), totaling 6,528 measures of spirometry (average of 55 measures per subject). An additional 61 subjects had spirometry values obtained exclusively after severe CFLD diagnosis. Taken together with the Pre-/Post- cohort, this constitutes the Post cohort (n = 179). The distribution of age at severe CFLD diagnosis for both cohorts is shown in S1 Fig. The average age at CFLD diagnosis for the Pre-/Post- and Post cohorts was 13.3 ± 4.7 years and 11.7 ± 5.2, respectively (Table 1). Spirometry values were collected over a median 4.3 years (range 0.3–16.8) before CFLD diagnosis, and a median 8.0 years (range 0.2–29.0) after diagnosis, totaling 8,249 FEV_1_ values informing the slopes of lung function decline model determined by our model (Table 1).

**Table 1:** Characteristics of CF subjects with severe liver disease (CFLD) and spirometry testing.

Description		Lung Function data sets	
		Pre-/Post- cohort	Post cohort
CFLD subjects (*n*)		118	179
	Male subjects (%)	61	62
*CFTR* genotype, *n* (%)			
	Phe508del/Phe508del	66 (56)	105 (58)
	Phe508del/Other	46 (39)	66 (36)
	Other/Other	6 (5)	11 (6)
Total spirometry tests analyzed (*n*)		6,528	6409
	Pre-diagnosis/Post-diagnosis (*n*)	1840/4688	0/6409
CFLD diagnosis			
	Mean age (±SD) in yrs	13.3 (± 4.7)	11.7 (± 5.2)
	Median (range) in yrs <6 (%) 6–11 (%) 12–17 (%) >18 (%)	12 (7–28) 0 44 37 19	11 (0–28) 11 43 32 14
Spirometry test Information			
	Mean (±SD) # spirometry/patient	55 (± 27)	36 (± 27)
	Median (range) # spirometry/patient	54 (5–145)	29 (1–148)
	Median (range) # yrs of spirometry	4.3 (0.3–16.8)/7.3 (0.2–20.3)	8.0 (0.2–29.0)

“Post” cohort contains all CFLD patients from the “Pre-/Post-” cohort as well as additional severe CFLD patients with FEV_1_ measures obtained only after severe CFLD diagnosis.

**Fig 1:**
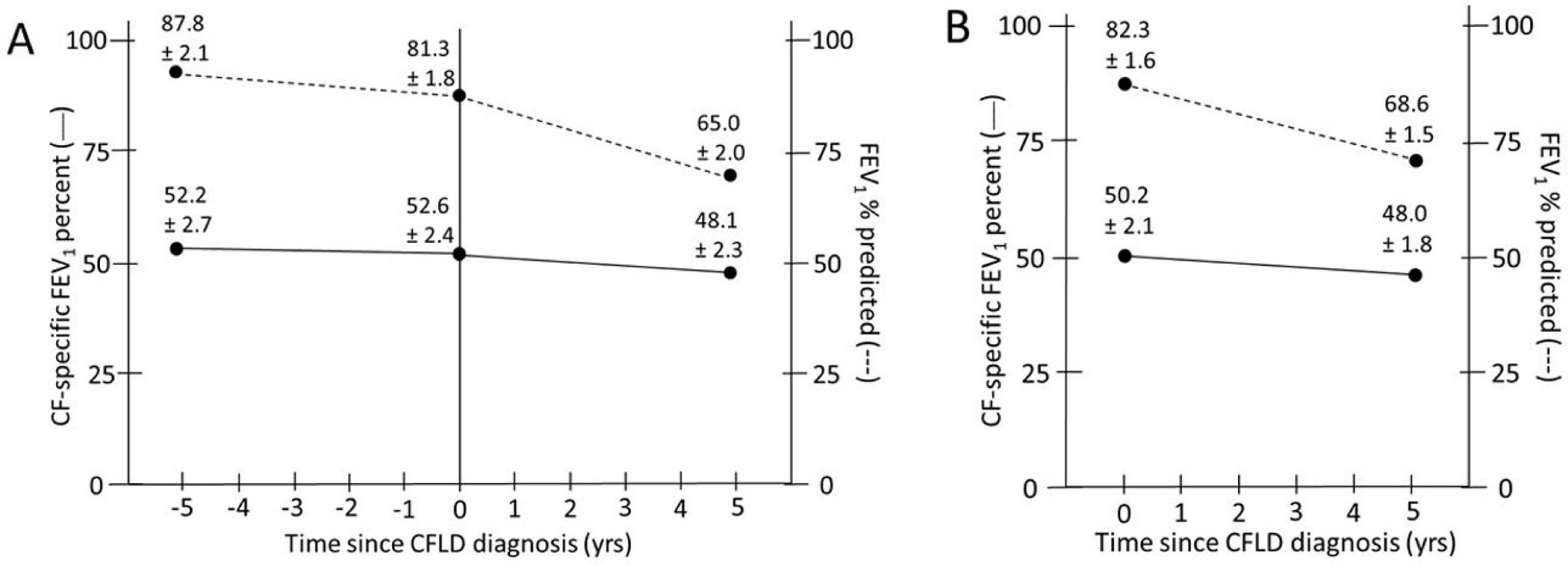
Plot of average slope decline in lung function in CFLD subjects. (**A**) Points are estimated mean values (±SE), of outcome plotted at t = -5 years (before CFLD diagnosis), t = 0 years (time of CFLD diagnosis), and t = 5 years (after CFLD diagnosis) for a patient age 13.3 years (average age at CFLD diagnosis). CF-specific FEV_1_ percentile (solid line) and FEV_1_% predicted (dashed line) are shown in 118 CFLD subjects with lung function values before and after CFLD diagnosis. (**B**) Averaged data at t = 0 and t = 5 years (±SE), including an additional 61 patients with post-CFLD diagnosis lung function values (total subjects, n = 179).

To analyze the relationship of lung function and severe CFLD in a longitudinal manner before and after severe CFLD diagnosis, we examined the slope of lung function decline relative to both a normal (non-CF, healthy) reference population (FEV_1_% predicted) and a CF-specific reference population (CF-specific FEV_1_ percentile: matched for gender, age and height) (Table 2). Model representation is shown for a ten-year timespan, five years before and five years after diagnosis of CFLD using the average age of CFLD diagnosis. In a linear mixed model using lung function measures from the Pre-/Post- cohort, the mean FEV_1_ five years prior to severe CFLD was 87.8 ± 2.1% predicted when referenced to a normal population (Fig 1A). When we referenced the severe CFLD FEV_1_ values to a CF population, the mean FEV_1_ five years prior to CFLD diagnosis was in the 52^nd^ percentile (or average for CF patients) (Fig 1A). The FEV_1_% predicted values demonstrated a negative slope with lung function declining before (-1.31% predicted/yr, 95% CI -2.03, -0.58) and after (-3.25% predicted/yr, 95% CI -3.82, -2.69) CFLD diagnosis (Table 2; Fig 1A). These slopes of lung function over time in severe CFLD were significantly different from zero both before and after CFLD diagnosis (p = 0.0004 and p<0.0001, respectively). Further, lung function decline following CFLD diagnosis was significantly worse than lung function decline prior to CFLD diagnosis (difference in slopes -1.94% predicted/yr, 95% CI -2.88, -1.00; p<0.0001) (Table 2; Fig 1A).

**Table 2:** Mixed linear model results of lung function slope decline in CFLD.

Description		Estimate ± SE (95% CI)	p-value
**Compared to Healthy Population (FEV**_**1**_**% predicted) matched for gender, age, & height**			
n = 118 CFLD subjects (Pre-/Post- cohort)			
	pre-CFLD slope (% predicted/year)	-1.31 ± 0.37 (-2.03, -0.58)	0.0004
	post-CFLD slope (% predicted/year)	-3.25 ± 0.29 (-3.82, -2.69)	<0.0001
	Difference in slopes (post-pre)	-1.94 ± 0.48 (-2.88, -1.00)	<0.0001
	FEV1 at age of CFLD diagnosis	81.3 ± 1.8 (77.8, 84.8)	
n = 179 CFLD subjects (Post cohort)			
	post-CFLD slope (% predicted/year)	-2.74 ± 0.23 (-3.18, -2.30)	<0.0001
	FEV_1_ at age of CFLD diagnosis	82.3 ± 1.6 (79.2, 85.3)	
**Compared to CF Population (CF-specific FEV**_**1**_ **percentile) matched for gender, age, & height**			
n = 118 CFLD subjects (Pre-/Post- cohort)			
	pre-CFLD slope with average age of CFLD diagnosis (% predicted/year)	-0.08 ± 0.50 (-0.90, 1.06)	0.8735
	post-CFLD slope with average age of CFLD diagnosis (% predicted/year)	-0·91 ± 0·33 (-1·56, -0·25)	0.0064
	Difference in slopes (post-pre)	-0.99 ± 0.64 (-2.25, 0.28)	0.1268
	FEV_1_ at age of CFLD diagnosis	52.6 ± 2.4(48.0, 57.3)	
n = 179 CFLD subjects (Post cohort)			
	post-CFLD slope (% predicted/year)	-0.45 ± 0.27 (-0.97, 0.07)	0.0902
	FEV_1_ at age of CFLD diagnosis	50.2 ± 2.1 (46.2, 54.2)	

Slope of lung function measures pre- and post-CFLD diagnosis were analyzed independently, then compared with each other in n = 118 subjects. In n = 179 subjects, slope of lung function decline is analyzed post-CFLD diagnosis only.

For each analysis, p<0.05 indicates a mean slope or difference in mean slopes significantly different from zero.

However, when FEV_1_ was referenced as CF-specific FEV_1_ percentile values (calculated referencing a large CF population), values after CFLD diagnosis compared to those before CFLD diagnosis suggested no difference in slope of lung function over time (-0.99% predicted/yr, 95% CI -2.25, 0.28; p = 0.1268) (Table 2; Fig 1A). We further included the FEV_1_ measurements of an additional 61 patients with spirometry measures (n = 1,721) recorded exclusively after severe CFLD diagnosis (n = 179 subjects; Table 2; Fig 1B). In 179 patients with severe CFLD, the average CF-specific FEV_1_ percentile at severe CFLD diagnosis was 50.2 ± 2.1 percentile, and five years after diagnosis was 48.0± 1.8 percentile. This large cohort of severe CFLD patients suggested the slope of CF-specific FEV_1_ percentile after severe CFLD diagnosis did not differ from zero (-0.45% predicted/yr, 95% CI -0.97, 0.07; p = 0.0902; Table 2; Fig 1B). While there is a modest trend toward significance (p = 0.0902), the analysis of our data yields slope constants that do not vary (and thus the difference from zero does not further approach significance) even with increased years of analysis under the model.

## Discussion

We studied a large cohort of stringently phenotyped CF subjects with an extreme form of liver disease (severe “CFLD”; cirrhosis with portal hypertension) to rigorously test for association of CFLD with lung function decline in CF. Because lung function decline is recognized to be greatest during the adolescent age in CF [21], referencing the slope of lung function decline in severe CFLD to that of decline in the general CF population was critical. Using 8,249 measures of FEV_1_ from 179 severe CFLD subjects, and a mixed-effects linear model in statistical analysis, we indicate that severe CFLD is not associated with an adverse effect on lung disease severity or decline in lung function when compared to the lung function values of a broad CF population. Indeed, analysis of FEV_1_ measures pre- and post- severe CFLD diagnosis across this large cohort demonstrates that mean lung function, and lung function decline, in severe CFLD patients are approximately at average for CF patients without CFLD. Our findings demonstrate a significant decline in lung function in severe CFLD patients referenced to a healthy population (FEV_1_% predicted). However, CF-specific FEV_1_ percentiles are at approximately the 50^th^ percentile at baseline, and remain stable, when referenced to the general CF population, indicating a similar rate of lung function decline in non-CFLD and severe CFLD subjects. Unique to our study, we calculated CF-specific FEV_1_ by referencing FEV_1_ measures to age, gender, and height matched subjects in a United Sates population of 21,000 CF subjects [20].

We acknowledge several modest limitations of our study. First, in comparing lung function of patients with severe CFLD to that of the CF reference (Kulich) cohort, we recognize that as many as 3–5% of those CF reference patients may have severe CFLD, and ~10% will have “pancreatic sufficient” genotypes [22]. However, the magnitude (if any) of impact on the CF percentiles will be small and would not impact the calculated slope decline. We additionally recognize that the age of diagnosis of severe CFLD may lag behind occurrence of portal hypertension, however because our model was developed to assess lung function slope five years before and five years after diagnosis of CFLD, our model would detect impact on lung function in this surrounding period. Finally, we also recognize that our conclusions relate largely to CFLD patients without ascites, as only a small minority (6.7%) of our patients had ascites existing at the time of CFLD diagnosis. In principle, the presence of ascites may adversely impact lung function due to limited diaphragmatic excursion. However, our model of FEV_1_ trend over a five-year period following CFLD diagnosis showed that lung function remains at the average CF-specific FEV_1_ percentile, even with the inclusion of these most severe cases of CFLD with ascites.

In summary, our cross-sectional cohort study indicates that subjects with severe CFLD have lung function, and lung function decline, on par with age, gender, and height matched CF subjects in a large United States CF population. These findings suggest that there is no underlying genetic risk, neither protective nor detrimental, in severe CFLD subjects, for development of better (or worse) pulmonary disease relative to non-severe CFLD counterparts. The results of this study indicate that patients with severe CFLD fare neither better nor worse in lung disease severity or decline than a reference CF population. Despite this evidence, because severe liver disease, in itself, yields additional comorbidities including immunologic and hematologic complications (e.g., thrombocytopenia, hypercoagulability), conclusions regarding the overall prognosis in CFLD vs CF patients without liver disease cannot be drawn on the basis of pulmonary prognosis, or rates of lung function decline, alone. Indeed, such comorbidities in CFLD must be factored into the complex considerations for lung transplantation, as pulmonary disease remains the leading cause of morbidity and mortality in patients with CF. We conclude that young patients with severe CFLD demonstrate significant FEV_1_% predicted decline, a phenomenon which is already well known to occur in adolescents and young adults with CF [21], and we further indicate that this decline in severe CFLD subjects does not differ from a vast CF reference population. Thus, nationally recommended guidelines for CF pulmonary health management, including screening and chronic treatment of disease, should be considered in the care of all patients with CF independent of the presence, or absence, of liver disease.

## Supporting information

S1 Fig Distribution of age at CFLD diagnosis. “Post” cohort includes 179 CFLD subjects with known age of severe liver disease diagnosis (white bars). Inset of “Pre-/Post-” cohort (black bars) is comprised of a subset (n = 118) of CFLD subjects with lung function measures both before and after severe CFLD diagnosis.
(TIFF)

S1 Table Full name of sub-site and local approving IRBs. (DOCX)

S2 Table Lung function and age measurements. (XLSX)
