# Supplementary figures and images for: Analysis of a large cohort of cystic fibrosis patients with severe liver disease indicates lung function decline does not significantly differ from that of the general cystic fibrosis population
Source: PLoS One. 2018 Oct 11;13(10):e0205257. doi: 10.1371/journal.pone.0205257 (PMC6181334; doi:10.1371/journal.pone.0205257)

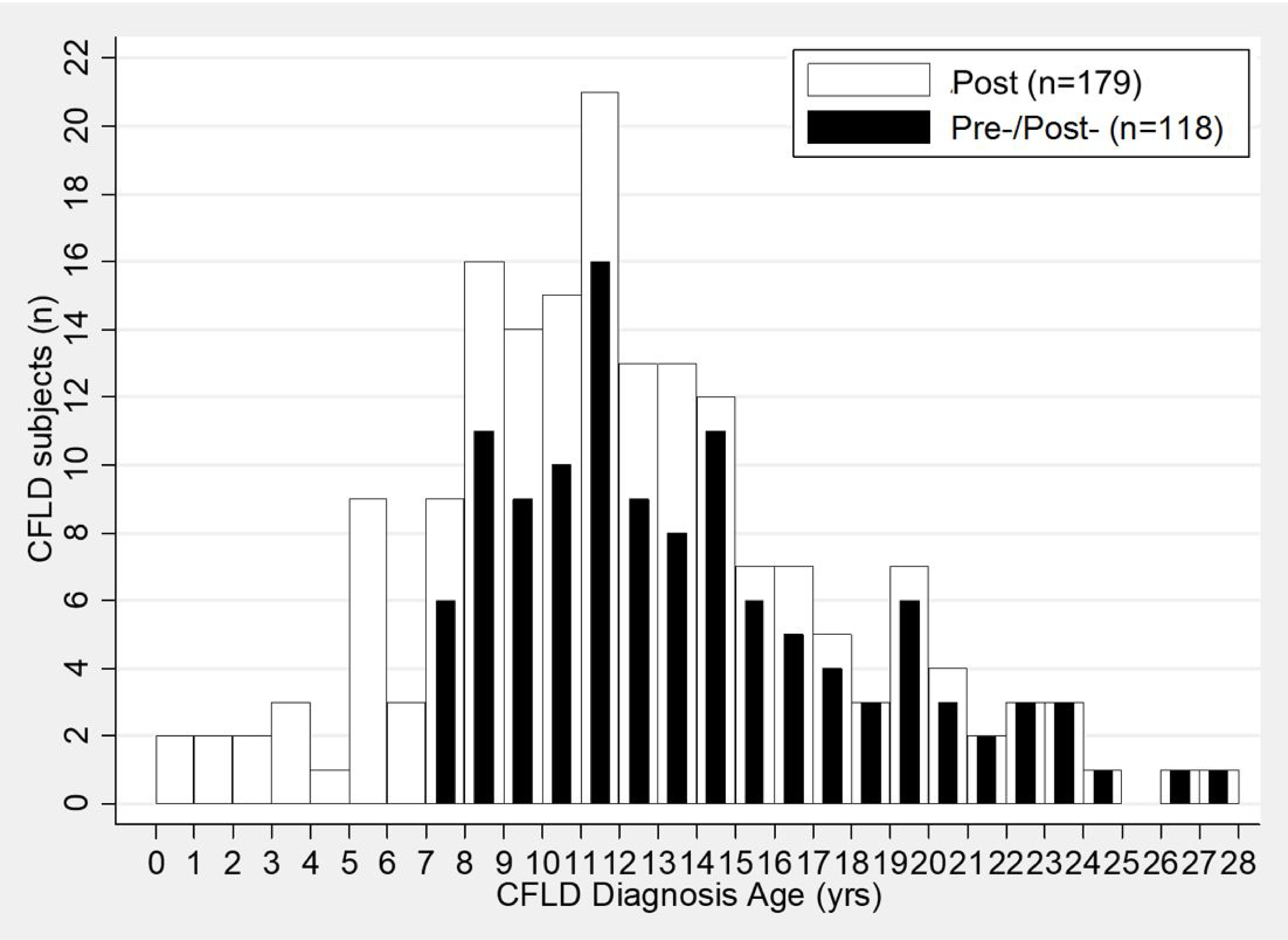

Supplement: S1 Fig — “Post” cohort includes 179 CFLD subjects with known age of severe liver disease diagnosis (white bars). Inset of “Pre-/Post-” cohort (black bars) is comprised of a subset (n = 118) of CFLD subjects with lung function measures both before and after severe CFLD diagnosis. (TIFF) [file pone.0205257.s001.tiff]
