# Supplementary material for: Analysis of a large cohort of cystic fibrosis patients with severe liver disease indicates lung function decline does not significantly differ from that of the general cystic fibrosis population
Source: PLoS One. 2018 Oct 11;13(10):e0205257. doi: 10.1371/journal.pone.0205257 (PMC6181334; doi:10.1371/journal.pone.0205257)
Supplement: S1 Table — (DOCX) [file pone.0205257.s002.docx]

**Supporting Information**

**S1 Table: Full name of sub-site and local approving IRBs**

1. Case Western Reserve University; Office of Research and Technology Management
2. Children's Hospital Los Angeles; Human Subjects Protection Program
3. Children's Hospital of Chicago; Office of Research Integrity & Compliance
4. Cincinnati Children's; Office of Research Compliance and Regulatory Affairs
5. Dartmouth; Committee for the Protection of Human Subjects
6. Drexel University; Office of Research
7. Emory University; Institutional Review Board
8. Indiana University; Human Subjects & Institutional Review Boards
9. Medical College of Georgia; Augusta University Human Assurance Committee
10. Medical University of South Carolina; Institutional Review Board for Human Research
11. Mount Sinai Beth Israel; Program for the Protection of Human Subjects
12. Nationwide Children's; Institutional Review Board
13. Promedica; Institutional Review Board
14. Saint Louis University; Institutional Review Board
15. Seattle Children's Hospital; Institutional Review Board
16. State University of New York; Institutional Review Board
17. The Hospital for Sick Children (SickKids; CA); Research Ethics Board
18. University of Alabama Birmingham; Office of the IRB
19. University of Buffalo; Human Research Protection Program
20. University of Florida; Institutional Review Board
21. University of Iowa; Human Subjects Office
22. University of Michigan; Human Research Protection Program
23. University of Mississippi Medical Center; Human Research Office
24. University of Nebraska Medical Center; Institutional Review Board
25. University of North Carolina at Chapel Hill; IRB and Office of Human Research Ethics
26. University of Pittsburgh; Institutional Review Board
27. University of Tennessee Health Science Center; Institutional Review Board
28. University of Utah; Institutional Review Board
29. University of Virginia; Institutional Review Board for Health Sciences Research
30. Vanderbilt University; Human Research Protection Program
31. Via Christi Research; Institutional Review Board
32. Virginia Commonwealth University; Office of Research and Innovation
33. West Virginia University; Office of Research Integrity & Compliance
